# An *arsRB* resistance operon confers tolerance to arsenite in the environmental isolate *Terribacillus* sp. AE2B 122

**DOI:** 10.1093/femsec/fiab015

**Published:** 2021-01-29

**Authors:** Almudena Escobar-Niño, Leyre Sánchez-Barrionuevo, José Miguel Torres-Torres, Rafael Clemente, Gabriel Gutiérrez, Encarnación Mellado, David Cánovas

**Affiliations:** Department of Genetics, Faculty of Biology, University of Seville, Seville, 41012, Spain; Department of Microbiology and Parasitology, Faculty of Pharmacy, University of Seville, Seville, 41012, Spain; Department of Genetics, Faculty of Biology, University of Seville, Seville, 41012, Spain; Department of Microbiology and Parasitology, Faculty of Pharmacy, University of Seville, Seville, 41012, Spain; Department of Genetics, Faculty of Biology, University of Seville, Seville, 41012, Spain; CEBAS-CSIC, Campus Universitario de Espinardo, Murcia, 30100, Spain; Department of Genetics, Faculty of Biology, University of Seville, Seville, 41012, Spain; Department of Microbiology and Parasitology, Faculty of Pharmacy, University of Seville, Seville, 41012, Spain; Department of Genetics, Faculty of Biology, University of Seville, Seville, 41012, Spain

**Keywords:** arsenic, *Terribacillus*, *ars* genes, *arsB*, *arsC*, metal resistance

## Abstract

*Terribacillus* sp. AE2B 122 is an environmental strain isolated from olive-oil agroindustry wastes. This strain displays resistance to arsenic, one of the most ubiquitous carcinogens found in nature. *Terribacillus* sp. AE2B 122 possesses an unusual *ars* operon, consisting of the transcriptional regulator (*arsR*) and arsenite efflux pump (*arsB*) but no adjacent arsenate reductase (*arsC*) locus. Expression of *arsR* and *arsB* was induced when *Terribacillus* was exposed to sub-lethal concentrations of arsenate. Heterologous expression of the *arsB* homologue in *Escherichia coli*  *∆arsRBC* demonstrated that it conferred resistance to arsenite and reduced the accumulation of arsenic inside the cells. Two members of the *arsC*-like family (Te3384 and Te2854) found in the *Terribacillus* genome were not induced by arsenic, but their heterologous expression in *E. coli ∆arsC* and *∆arsRBC* increased the accumulation of arsenic in both strains. We found that both Te3384 and Te2854 slightly increased resistance to arsenate in *E. coli ∆arsC* and *∆arsRBC*, possibly by chelation of arsenic or by increasing the resistance to oxidative stress. Finally, arsenic speciation assays suggest that *Terribacillus* is incapable of arsenate reduction, in agreement with the lack of an *arsC* homologue in the genome.

## Teaser

*Terribacillus* harbors an unusual ars operon that confers resistance to arsenite in *Escherichia coli*.

## INTRODUCTION

Arsenic is a naturally occurring element classified as a class I carcinogen by the International Agency of Research on Cancer. Arsenic contamination is derived from both anthropogenic sources, such as industrial waste or agricultural practices, and natural sources, through processes such as dissolution of arsenic-containing minerals (Hettick *et al*. 2015). Inorganic arsenic can appear in different oxidation states: -3 (arsine, AsH_3_), 0 (metallic, As^0^), +3 (arsenite, AsO_3_^3−^) and +5 (arsenate, AsO_4_^3−^), depending on the environmental conditions. Arsenate is the predominant form in soil and surface water, while arsenite prevails under reducing conditions in anaerobic groundwater. Both forms exist in terrestrial and aquatic environments independently of the pH and the redox potential, since the redox chemical reactions between arsenate and arsenite are relatively slow (Zhu, Xue and Kappler 2017). Although arsenite is more toxic than arsenate, the reduction of the oxidized form into arsenite is part of one of the most widespread resistance mechanisms of bacteria to this metalloid (Rosen 2002; Paez-Espino *et al*. 2009). This reduction has also been described as a common process for solubilizing arsenic from minerals, which results in the contamination of water supplies (Zhu, Xue and Kappler 2017). Additionally, some prokaryotes, such as *Chrysiogenes arsenatis*, *Shewanella* sp. strain ANA-3 and *Bacillus selenitireducens*, can utilize arsenate as a terminal electron acceptor for anaerobic respiration, expressing a respiratory reductase (*arrAB*), which is also considered to be involved in arsenic release from anaerobic sediments worldwide (Ahmann *et al*. 1994; Dowdle, Laverman and Oremland 1996; Saltikov and Newman 2003; Tsuchiya *et al*. 2019; Mazumder *et al*. 2020).

Bacteria have developed a great variety of mechanisms to avoid the toxicity of arsenic, e.g. (i) peroxidation reactions by membrane lipids, (ii) minimizing the absorption of arsenate by the phosphate uptake system, and (iii) the arsenic microbial detoxification pathway, which involves the *acr* and *ars* operons that are widespread in bacteria (Paez-Espino *et al*. 2009; Ben Fekih *et al*. 2018; Dunivin, Yeh and Shade 2019; Garbinski, Rosen and Chen 2019). The minimal constituents of the *ars* operon are three genes organized into a single transcriptional unit: *arsR*, which encodes the arsenic transcriptional repressor; *arsB*, which encodes a membrane-bound arsenite permease that exports arsenite; and *arsC*, which encodes a reductase that converts arsenate into arsenite (Rosen 1999, 2002; Paez-Espino *et al*. 2009). *arsB* is widespread in the chromosomes of Actinobacteria, Firmicutes and Proteobacteria (Dunivin, Yeh and Shade 2019). More complex *ars* operons have also been described in bacteria, with additional genes encoding an ATPase (ArsA), an arsenite chaperon (ArsD), an organoarsenical oxidase (ArsH) and the arsenite methyltransferase (ArsM) (Qin *et al*. 2006; Paez-Espino *et al*. 2009; Chen, Bhattacharjee and Rosen 2015; Ben Fekih *et al*. 2018; Paez-Espino *et al*. 2020).

The addition of composted olive oil waste has been shown to stimulate soil microbial communities, reducing their stress and increasing their growth, activity and functional diversity. Addition of composted olive oil waste tends to reduce the solubility of trace elements in the amended soils, in general. However, in the particular case of arsenic, different studies have reported the mobilization of arsenic after the addition of olive oil waste compost to trace elements-contaminated soil (Clemente *et al*. 2015). Additionally, over the past few decades there has been a growing interest in utilizing biomass from trace element-contaminated lands to produce bio-energy (Dastyar *et al*. 2019). *Terribacillus* sp. AE2B 122 is an environmental strain isolated from olive-oil agroindustry wastes able to perform transesterification reactions of lipids (Escobar-Nino *et al*. 2014). This capacity can be used for the production of biofuels, and indeed, this strain was one of the top five biofuel producers found in that study. This bacterium is included in group 1 of the genus *Bacillus*, together with many other halotolerant and halophilic genera (Escobar-Nino *et al*. 2014). This genus was reported for the first time in 2007 (An *et al*. 2007). Until now, only the genome sequences of *Terribacilus aidingensis* MP602 (Lu *et al*. 2015) and *Terribacillus saccharophilus* (Kalinowski *et al*. 2018) have been available. Here, we show that *Terribacillus* sp. AE2B 122 displays resistance to arsenite and arsenate, but shows an unusual *ars* gene organization cluster, lacking an arsenate reductase. A homologue of the arsenite pump *arsB* is functional in *Terribacillus* sp. AE2B 122, and can promote resistance to arsenite in *E. coli* by reducing the intracellular arsenic accumulation.

## MATERIALS AND METHODS

### Strains, media and culture conditions

The arsenic-resistant bacterium, *Terribacillus*sp. AE2B 122 was isolated from ponds containing liquid wastes located in an olive oil mill in Ecija (Sevilla, Spain) (Escobar-Nino *et al*. 2014). The *Escherichia coli* strains used and constructed in this study are listed in Table S1. *Terribacillus*sp. AE2B 122 was grown in PYA (peptone 1%, yeast extract 0.5%, K_2_HPO_4_ 0.1%, MgSO_4_.7H_2_O 0.02%) + 1.5% NaCl media at 30°C. *Escherichia coli* strains were grown in tryptone 10 g/L, yeast extract 5 g/L, NaCl 10 g/L (LB) media at 37°C. Tetracyclin, gentamicin and/or isopropyl β-d-1-thiogalactopyranoside (IPTG) were added when required.

### Whole-genome sequencing of *Terribacillus* sp. AE2B 122

The sequencing was performed by BGI-HongKong Co., Ltd., using Illumina technology and a strategy of 2 × 90 bps. Sequences were first filtered to eliminate low quality sequences, and then the oligonucleotide sequences were eliminated. The SOAP *de novo* algorithm version 1.05 was used (Li *et al*. 2008). The results were assembled locally and optimized according to the paired ends and the overlapping relationships, mapping the readings to build contigs and supercontigs. In-house automatic annotation was carried out in our laboratory using the RAST server, which performs rapid annotations using subsystem technology (Aziz *et al*. 2008).

### Phylogenetic analysis

A search using the Batch web CD-search Tool (Marchler-Bauer *et al*. 2010, 2016) was conducted to select the homologous Ars proteins to be included in the study. Multiple alignments were made with Clustal Omega (Sievers *et al*. 2011). Maximum-likelihood trees were generated using the IQ-Tree server (Trifinopoulos *et al*. 2016). Branch support analysis was evaluated by 1000 ultrafast bootstrap replicates and by the SH-aLRT test. ModelFinder, implemented at the IQ-Tree server, was used to find the free rate heterogeneity substitution model that best fit the alignments (Kalyaanamoorthy *et al*. 2017). The online tool iTOL was used to edit the constructed phylogenetic trees (Letunic and Bork 2016).

### Heterologous expression of *ars* homologues in *E. coli*

To express the putative *arsB* and *arsC* homologues of *Terribacillus* sp. AE2B 122 in *E. coli ars* mutants under the control of the *lac* promoter, the plasmids pTe3675, pTe2733, pTe3384 and pTe2854 were constructed using specific primers (Table S2, Supplementary data). To construct pTe3675 and pTe2733, the corresponding genes were amplified by PCR from *Terribacillus* sp. AE2B 122 DNA using the primers MAO393 and MAO394 (for Te3675) and MAO398 and MAO399 (for Te2733). *Eco*RI-*Knp*I fragments were ligated into the vector pVLT31 (Cases, de Lorenzo and Pérez-Martín 1996) linearized with the same enzymes. *Escherichia coli* DH5α strain was transformed and selected on LB plates supplemented with tetracycline. To check the construct, we used the primers MAO160 and MAO173 (Te3675), and MAO160 and MAO173 (Te2733) for sequencing.

A similar strategy was followed for the construction of pTe3384 and pTe2854, using the primers MAO403 and MAO404, and MAO406 and MAO407, respectively. The PCR fragments were cloned into pIZ1016 (Martínez-Pérez *et al*. 2004) using *Eco*RI-*Xba*I sites. Positives clones were confirmed by sequencing using the primers MAO160 and MAO404 (Te3384), and MAO160 and MAO407 (Te2854) primers.

### Arsenic resistance assays in *E. coli* strains and *Terribacillus* sp. AE2B 122

Overnight cultures of *Terribacillus* sp. AE2B 122 and *E. coli* were adjusted to a final OD_600_ of 0.5. Ten µl of the final dilution were streaked onto PYA + NaCl solid medium supplemented with 10 mM arsenate or 5 mM arsenite, containing 10 µM IPTG, 1 mM IPTG or 1% glucose. Plates were incubated at 30°C for 1–3 days. PYA + NaCl plates without arsenic were used as the negative control.

Overnight cultures of *Terribacillus* sp. AE2B 122 and *E. coli* strains were diluted to a final OD_600_ of 0.05. Ten µl of each strain dilution were mixed with 140 µl of PYA + NaCl medium (*Terribacillus* sp. AE2B) or LB (*E. coli* strains) containing increasing concentrations of arsenate (2.5, 5 or 10 mM) or arsenite (1, 2.5 or 5 mM). Those cultures were grown in triplicate in 96-well microtiter plates at 37°C with shaking. Growth was monitored by measuring the absorbance at 600 nm every hour for 24 h. Strains growing in PYA + NaCl medium (*Terribacillus* sp. AE2B 122) or LB (*E. coli*) without arsenic were used as the negative control.

### RT-qPCR analysis

*Terribacillus* sp. AE2B 122 was grown in PYA + NaCl and induced for 6 h with 0, 0.375 or 1.5 mM arsenate. Total RNA was isolated using Trizol method according to the protocol of the manufacturer and quantified with a NanoDrop (Thermo Fisher Scientific). To eliminate genomic DNA, RNA was incubated with RNase-free DNase (Promega) according to the instructions of the manufacturer. After treatment, the samples were adjusted to 12.5 ng μl^−1^ and employed in RT-qPCR reactions with One Step SYBR PrimeScript RT-PCR kit (Takara Bio Inc.). Primers used in the RT-qPCR analysis were designed using the software Primer Express (Table S3, Supplementary data). Three independent biological replicates were analyzed for each sample using technical triplicates.

### Bacterial growth under oxidative stress

To check the resistance to oxidative stress, the *E. coli* cultures were adjusted to 10^7^ cell ml^−1^, and cells were diluted to concentrations of 10^6^, 10^5^ and 10^4^ cells ml^−1^ with distilled water, and spotted (2 μl) onto LB solid medium added with 1 mM IPTG and H_2_O_2_. The inoculated plates were incubated at 37°C for 2 days. LB plates without H_2_O_2_ were used as the negative control.

### Accumulation of arsenic in *E. coli*

The *E. coli* strains W3110, WC3110, AW3110, AW3110/pTe2854, AW3110/pTe3384, AW3110/pTe3675, WC3110/pTe2854 and WC3110/pTe3384 were pre-cultured overnight. Then, 0.6 ml of each overnight culture was used to inoculate Erlenmeyer flasks (250 ml) containing 60 ml of LB medium supplemented with 1 mM arsenate or 3 mM arsenite. The inoculated flasks were incubated with continuous stirring (200 rpm) for 5 h at 37°C. After incubation, a volume of 15 ml of culture was filtered through nitrocellulose filters (0.45-μm pore diameter; Whatman) and the cells retained on the filters were washed twice with 0.05 M potassium phosphate buffer (pH 7.6). Filtering and washing were performed using a vacuum pump (Millipore 230V). The filters, which contained the retained cells, were treated with 10 ml of 65% nitric acid at 70°C for 120 min. Nitric acid (Sigma-Aldrich) was doubly distilled from 65% HNO_3_ reagent grade with a DST-1000 sub-boiling acid purification system from Savillex (Savillex, Eden Prairie, MN, USA) and diluted with Milli-Q water. Samples were analyzed in an Agilent 8800 ICP-MS/MS (Agilent Technologies) at the General Facilities for Research of the University of Sevilla (http://citius.us.es).

### Arsenic speciation

*Terribacillus* sp. AE2B 122 was pre-cultured overnight. Then, 2 ml of each overnight culture was used to inoculate 10 ml of PYA + NaCl medium and further incubated with continuous stirring (200 rpm) for 90 min at 30°C. Arsenate or arsenite were added to a final concentration of 0.9 mM and incubation continued with continuous stirring (200 rpm) at 37°C for 0, 15, 30 and 60 min. Next, 1.5 ml of culture was withdrawn at each time point and centrifuged for 1 min at 20 238 g, separating cells (intracellular) and supernatant (extracellular). The cells were first washed with 1 ml PYA + NaCl and finally resuspended in 70% MeOH. Arsenic speciation in *E. coli* strains was essentially performed following the same strategy with the only difference that 1 mM IPTG was added to the cultures to induce the expression of Te2854 and Te3384. Intracellular and extracellular fractions were stored at -20°C. Supernatant samples were directly diluted in mQ water, filtered through a 0.22 µm membrane filter and analyzed for major arsenic species (arsenite [AsIII], dimethylarsinic acid [DMA], monomethylarsonic acid [MMA] and arsenate [AsV]) by high-performance liquid chromatography ([HPLC]; Agilent 1260 Infinity with a PSAC1-PRPX 100SAX 250 mm x 4.6 mm column) coupled to hydride generation atomic fluorescence spectrometry (HG-AFS; PS Analytical 10.055 Millennium Excalibur). MeOH extracts were sonicated for 20 min and homogenized by vortexing. Aliquots of 0.05 ml were taken for total arsenic determination after digestion with concentrated HNO_3_ (4 ml) at 70°C (2 h), taken to a final volume of 10 ml and finally filtered through 0.45 membrane filters before analysis by HG-AFS. The remaining MeOH extracts were centrifuged (7 min, 15 000 rpm) and aliquots of 0.05 ml were taken to a final volume of 1 ml with mQ water, and filtered through 0.22 membrane filters before arsenic speciation analysis by HPLC-HG-AFS. The species were separated using an isocratic mobile phase with 20 mM phosphate buffer at pH 6.25. The recovery of arsenate and arsenite from the cells was estimated to be 136% on average.

### Nucleotide sequence accession numbers

The nucleotide sequence of the genome is deposited under accession number PRJEB33524 in the European Nucleotide Archive and NCBI (GenBank: CABVGR000000000.1). The nucleotide sequences of Te3675 (*arsB*) and Te3676 (*arsR*) were deposited under accession numbers MN068029 (BankIt2234525 ArsB) and MN068030 (BankIt2234525 ArsR), respectively, in the GenBank database (https://www.ncbi.nlm.nih.gov/genbank/).

## RESULTS

### Arsenic resistance in *Terribacillus* sp. AE2B 122

**Figure 1.**
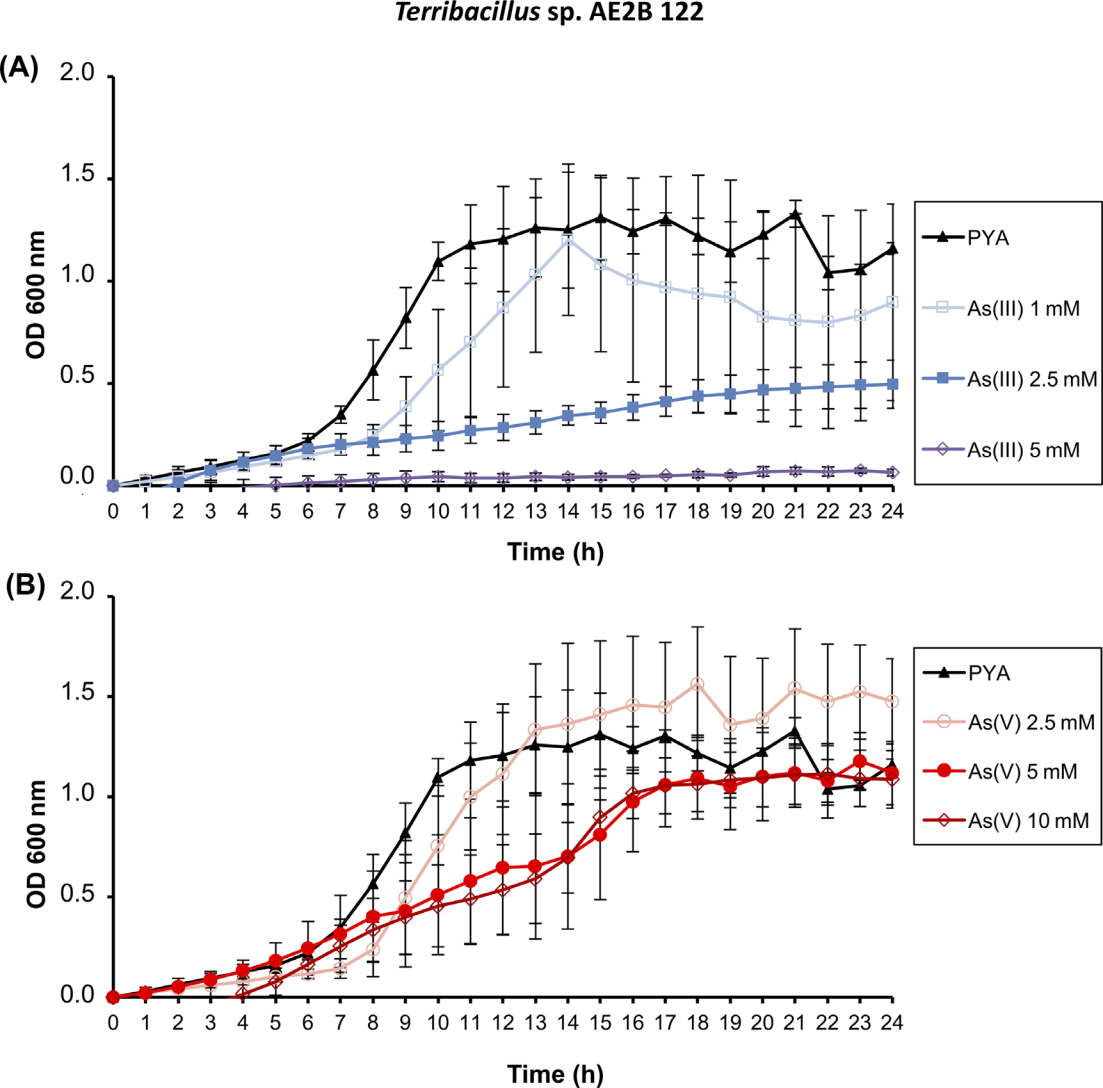
Arsenic resistance assays of *Terribacillus* sp. AE2B 122. *Terribacillus* sp. AE2B 122 was inoculated in PYA + 1.5% NaCl with the indicated concentrations of **(A)** arsenite, As(III) or **(B)** arsenate, As(V), at 37°C. Data shown are the mean of at least three independent experiments performed in technical duplicates ± standard errors of the mean (SEM).

In order to test tolerance to arsenate and arsenite, *Terribacillus*sp. AE2B 122 was grown in PYA + NaCl medium in the presence of different concentrations of these compounds and absorbance was monitored every hour for 24 h (Fig. 1). *Terribacillus* sp. AE2B 122 was more sensitive to arsenite than arsenate. This strain tolerated a concentration of 1 mM arsenite (Fig. 1A) and showed very slight growth at 2.5 mM arsenite. The tolerance to arsenate was higher, as is usually the case in other bacteria, with growth still observed at 10 mM (Fig. 1B).

### *Terribacillus* sp. AE2B 122 genome sequencing and bioinformatic analysis

The genome sequence of *Terribacillus* sp. AE2B 122 was assembled into 91 contigs and 22 supercontigs using the SOAP de novo program, to give a total genome size of 3718 326 bps (accession number PRJEB33524). The genome was annotated automatically using the RAST server and found to contain a total of 3769 open reading frames (ORFs). Among these annotated ORFs we found eight homologues of genes related to arsenic resistance. Two of these ORFs are homologues of *arsB* (Te2733 and Te3675), two are homologues of *arsC* (Te3384 and Te2854) and four are homologues of *arsR* (Te2310, Te3140, Te3676 and Te3724). BLAST searches using several *ars* genes and proteins (*arsH, arsM, arsR, arsC, arsB, arsD, arsI* and *arsP*) from reference strains did not reveal additional *ars* homologues in the genome of *Terribacillus* sp. AE2B 122. In addition, we also found homologues of genes involved in resistance to several metals: (i) eight homologues of chromate resistance genes, such as *chrA* (Viti *et al*. 2014) and nitroreductases, which may have secondary chromate reductase activity (Viti *et al*. 2014); (ii) seven homologues of Co-Zn-Cd resistance genes, including *zntR* (Nies & Silver 1995) and *czcD* (Moore *et al*. 2005); (iii) five homologues of cobalt resistance genes, such as *corA* and *corC* (Smith *et al*. 1998; Sponder *et al*. 2010); and (iv) five homologues of copper resistance genes, including *cutC* (Gupta *et al*. 1995), *csoR* (Marcus *et al*. 2016), *copA* and *copZ* (Reyes *et al*. 2006).

### Characterization of the arsenic resistance genes in the genome of *Terribacillus* sp. AE2B 122

Out of the eight ORFs annotated as *ars* genes in the genome of *Terribacillus* sp. AE2B 122, only one homologue of the repressor *arsR* (Te3676) and one homologue of the arsenite pump *arsB* (Te3675) appeared contiguous in the genome. No *arsC* homologue was found in their proximity, and consequently this species did not show the typical minimum operon arrangement (*arsRBC*) found in many organisms with resistance to this metalloid (Kaur, Kamli and Ali 2011). Te3675 and Te3676 are located in a 12 kb contig. RAST annotation predicted that the genes located upstream of Te3676 are a hypothetical protein (Te3677), a Na^+^/H^+^ antiporter (Te3678), another hypothetical protein (Te3679), a sodium/solute symporter family protein (Te3680) and a glycerate kinase (Te3681). The genes located downstream of Te3675 are predicted to be a pyridine nucleotide-disulfide oxidoreductase NADH dehydrogenase (Te3674), a transcriptional regulator of the AraC family (Te3673), a hypothetical protein (Te3672) and a short-chain dehydrogenase/reductase (SDR) (Te3671).

The *arsR*-like genes (Te2310, Te3140, Te3676 and Te3724) encoded predicted proteins showing the highest level of identity to ArsR family transcriptional regulators (Table S4, Supplementary data). All of them showed the typical motif HTH_ARSR identified in this family of repressor proteins. Te3676p showed the best hit, with 100% identity to a member of the ARSR subfamily of winged helix-turn-helix transcriptional regulators from *T. saccharophilus* (WP_095220794.1) (Table S4, Supplementary data). It also displayed a significant level of identity to the metal sensing ArsR-SmtB repressor from *Bacillus subtilis* (NP_388414.1; 52.8% identity) and a DNA-binding transcriptional repressor ArsR from *E. coli* K12 (NP_417958.1; 41.8% identity).

The two homologues of *arsB* (Te2733 and Te3675) contain conserved domains typical of ArsB proteins, which belong to the ArsB_NhaD superfamily of permeases (cI21473). This family includes plasma membrane arsenite-efflux transporter proteins that pump arsenite from the cytosol to the extracellular medium in different microorganisms (Garbinski, Rosen and Chen 2019). Te3675 encodes a predicted protein of 432 amino acids that showed high levels of identity to the predicted ArsB permease of *T. saccharophilus* (WP_095220793.1; 99.8% identity), and to functional ArsB permeases of different bacteria, such as *Staphylococcus aureus* pI258 (WP 000153629; 68.2% identity) and *E. coli* plasmid R773 (WP_063116263.1; 55.2% identity) (Table S4, Supplementary data). Te2733 encodes a predicted protein of 452 amino acids that showed a high level of identity to a predicted ArsB protein from *T. saccharophilus* (WP_095218693.1; 99% identity), and functional ArsB proteins of *S. aureus* pI258 (WP 000153629; 30.4% identity) and *E. coli* plasmid R773 (WP_063116263.1; 28.8% identity) (Table S4, Supplementary data), but also with a putative Na^+^/H^+^ antiporter from *B. subtilis* (NP_391484.1, 56.4% identity).

**Figure 2.**
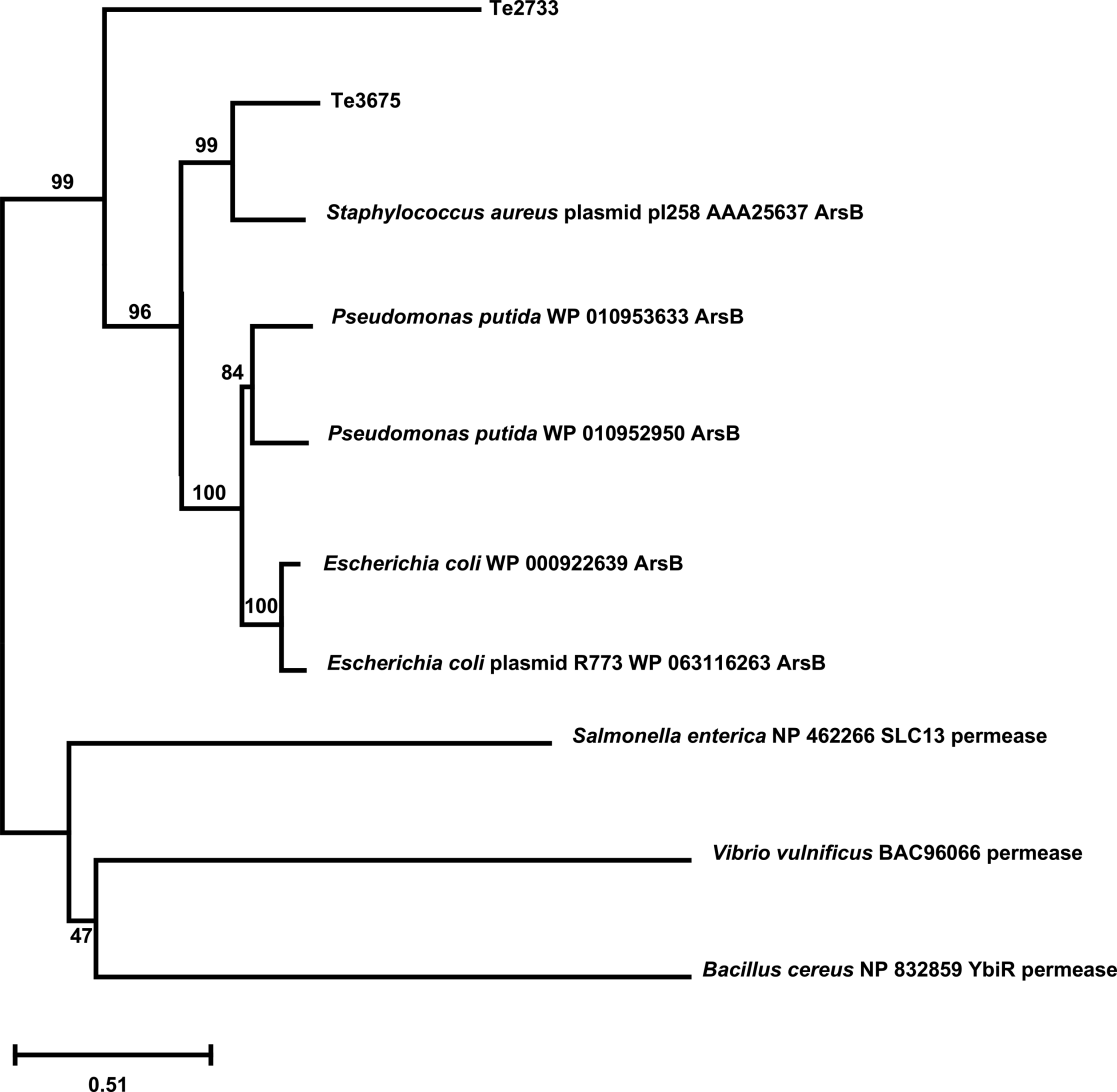
Phylogenetic reconstruction of the ArsB arsenite transporter homologues. The tree shows the phylogenetic relationships of eight representative transporter proteins of the ArsB_NhaD_permease superfamily (cI21473) and the two putative ArsB homologues (Te3675 and Te2733) of *Terribacillus* sp. AE2B. Maximum-likelihood trees were generated using the IQ-Tree server. Branch support analysis was evaluated by 1000 ultrafast bootstrap replicates. ModelFinder was used to find the free rate heterogeneity substitution model that best fit the alignment and online iTOl was used for tree editing.

Members of the ArsB family of permeases typically present 8–13 transmembrane helices. Te3675p with 12 predicted transmembrane helices and Te2733p with 13 predicted transmembrane helices fall within this range. Fig. 2 shows a phylogenetic tree depicting the relationship of both ArsB proteins (Te3675p and Te2733p) with other bacterial permeases classified into the four subgroups identified in the ArsB_NhaD_permease superfamily (cI21473). Although both proteins (Te3675p and Te2733p) seemed to be more closely related to members of the subgroup cd01118 (ArsB_permease), only Te3675p clustered together with ArsB proteins from Gram positive and Gram negative bacteria of that subgroup. Te2733p formed an outgroup from this cluster and was more distantly related to the members of this subgroup of ArsB proteins (yet it did not cluster with the other proteins of other groups in the ArsB_NhaD_permeases superfamily). The closest relative to Te3675p found in this analysis was ArsB of *S. aureus* plasmid pI258 (AAA25637).

The automatic annotation of the genome sequence by RAST predicted the presence of two *arsC* homologues (Te3384 and Te2854) scattered in the chromosome of the bacterium, which contained sequences similar to protein domains of the cd02977 ArsC_family (subfamilies: cd03033 ArsC_15kD; cd03034 ArsC_ArsC; cd03035 ArsC_Yfb; cd03032 ArsC_Spx; and cd3036 ArsC-like) belonging to the thioredoxin (TRX)-like superfamily (cI00388) (Table S4, Supplementary data). The genomic context of both genes did not provide any useful information about a putative function for these genes. Phylogenetic reconstruction revealed that Te2854p clustered together with proteins classified in the cd03032 ArsC_Spx subfamily and Te3384p was closely related to a protein from *Lactococcus lactis* (NP 266944) included in the cd03036 ArsC_like subfamily. None of the hypothetical ArsC-homologues of *Terribacillus* clustered together with the *bona fide E. coli* ArsC proteins (chromosomal and plamid R773) (Fig. S1 and Table S4, Supplementary data).

### Expression of arsenic resistance genes in *Terribacillus* sp. AE2B 122

**Figure 3.**
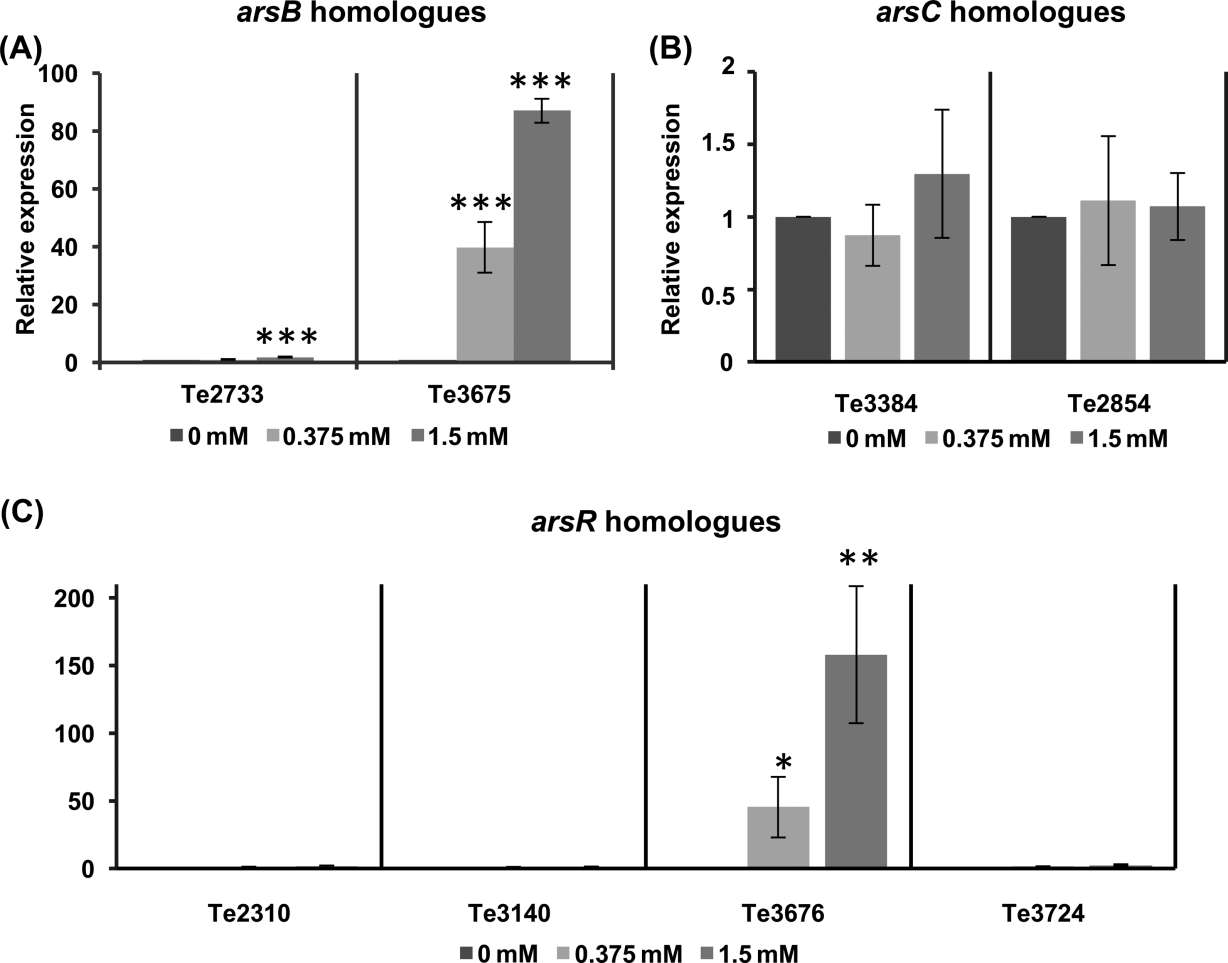
Gene expression of the *Terribacillus* sp. AE2B 122 *ars* homologues in response to arsenate induction. *Terribacillus* sp. AE2B 122 was grown in PYA and induced for 6 h with 0, 0.375 or 1.5 mM arsenate. The expression of the (**A**) *arsB* homologues (Te2733 and Te3675), (**B**) *arsC* homologues (Te3384 and Te2854) and (**C**) *arsR* homologues (Te2310, Te3140, Te3676 and Te3724) was quantified by RT-qPCR and normalized to the expression of the housekeeping gene *gyrA*. Data shown are relative to gene expression in media without arsenate (*n* = 3). Differences were considered to be significant in a Student's t-test: *P* ≤ 0.01 (***), *P* ≤ 0.02 (**) or *P* ≤ 0.05 (*).

RT-qPCR analysis was employed to quantify the expression levels of all the eight homologues of the *ars* genes in *Terribacillus* sp. AE2B 122 in the presence and absence of arsenate. Only Te3675 and Te3676 were inducible, showing an increase in expression when the concentration of arsenate in the culture media increased (Fig. 3). No induction was observed with the other homologue of *arsB* (Te2733) and the other homologues of*arsR*(Te2310, Te3140 and Te3724). None of the *arsC* homologues (Te3384 and Te2854) were induced by arsenate (Fig. 3B). Analysis of absolute gene expression without normalization to the control samples (i.e. in the absence of arsenate) revealed that Te2854 was expressed 10–20-fold higher than Te3384 (Fig. S2, Supplementary data).

### Complementation of an *ars* mutant of *E. coli* with the *Terribacillus* sp. AE2B 122 *ars* homologues

**Figure 4.**
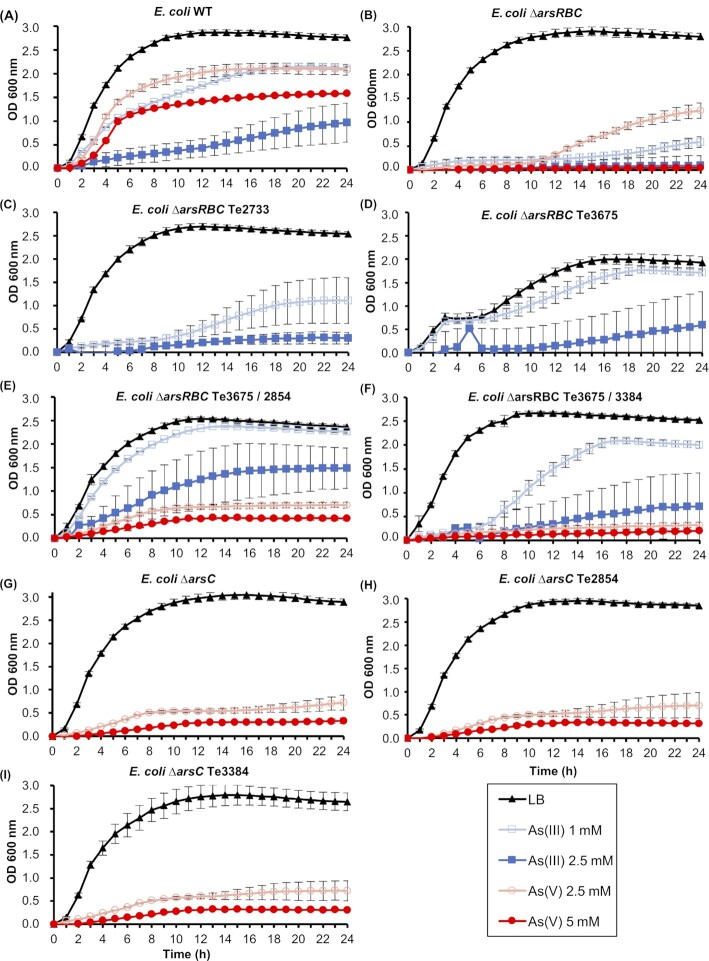
Arsenic resistance assays of *E. coli* W3110 (WT), *E. coli ∆arsRBC* and *E. coli ∆arsC* strains expressing the *arsB* and/or *arsC* homologues of *Terribacillus* sp. AE2B 122. (**A**) *E. coli*W3110 (wild type; *E. coli* WT); (**B**) *E. coli* AW3110 (*E. coli ΔarsRBC*); (**C**-**F**) *E. coli* AW3110 (*E. coli ΔarsRBC*) expressing the *arsB* homologue Te2733 (**C**) or Te3675 (**D**) or a combination of Te3675 with the *arsC* homologues Te2854 (**E) or Te3384 (**F); (**G**-**I**) *E. coli* WC3110 (*E. coli ΔarsC*) expressing the *arsC* homologue Te2854 (**H**) or Te3384 (**I**). The strains were inoculated in LB medium with 1 or 2.5 mM arsenite, As(III) or 2.5 or 5 mM arsenate, As(V), at 37°C. Data shown are the mean of at least three independent experiments performed in duplicates ± standard errors of the mean (SEM).

Heterologous expression experiments were performed to analyze the functional contribution of the putative *arsB* and *arsC* homologous genes from *Terribacillu*s sp. AE2B 122 to arsenate and arsenite resistance. First, we performed growth tests of *E. coli ars* mutants expressing the putative transporters, reductases or the four different combinations of the transporters and reductases. Te3675, but not Te2733, complemented the *∆arsB* mutation in *E. coli* AW3110 (*∆arsRBC*) and provided resistance to 1 mM arsenite and partial resistance to 2.5 mM arsenite (Fig. 4A-D). Similar results were obtained in the experiments performed on solid medium (Fig. S3, Supplementary data). This result is consistent with Te3675, but not Te2733, being inducible in *Terribacillus* sp. AE2B 122 (Fig. 3).

Since no other homologues of *arsC* were found in the genome of *Terribacillus* sp. AE2B 122, we also decided to test whether Te3384 and Te2854 could confer arsenate resistance to an *E. coli ∆arsC* mutant. Both Te3384 and Te2854 seemed to confer a slight resistance to 2.5 mM arsenate to *E. coli ∆arsC* in liquid media (Fig. 4A, G-I). This observation was independently confirmed on solid media in the case of Te3384 (but not of Te2854), which allowed the *E. coli ∆arsRBC* mutant to grow in 10 mM arsenate (Fig. S3, Supplementary data). When both the *arsB*-homologue Te3675, and either Te3384 or Te2854, were co-expressed in *E. coli ∆arsRBC* (Fig. 4E-F), the results obtained were similar to the expression of either gene individually, i.e. recovery of wild-type growth in arsenite (1 and 2.5 mM), and a slight increase in resistance to 2.5 and 5 mM arsenate, accounting for 12–20% of the wild-type growth under the same conditions. Co-expression of Te2733 and Te3384 or Te2854 did not improve the resistance of *E. coli ∆arsRBC* to arsenite or arsenate compared with expression of the single genes (Fig. S4, Supplementary data). Together, these data suggest that Te3675 provides the *E. coli ars* mutant with resistance to arsenite, and that Te3384 and Te2854 provide partial resistance to arsenate that is independent of the arsenite pump.

### Arsenic accumulation in *E. coli ars* mutants expressing the *Terribacillus* sp. AE2B 122 *ars* homologues

**Figure 5.**
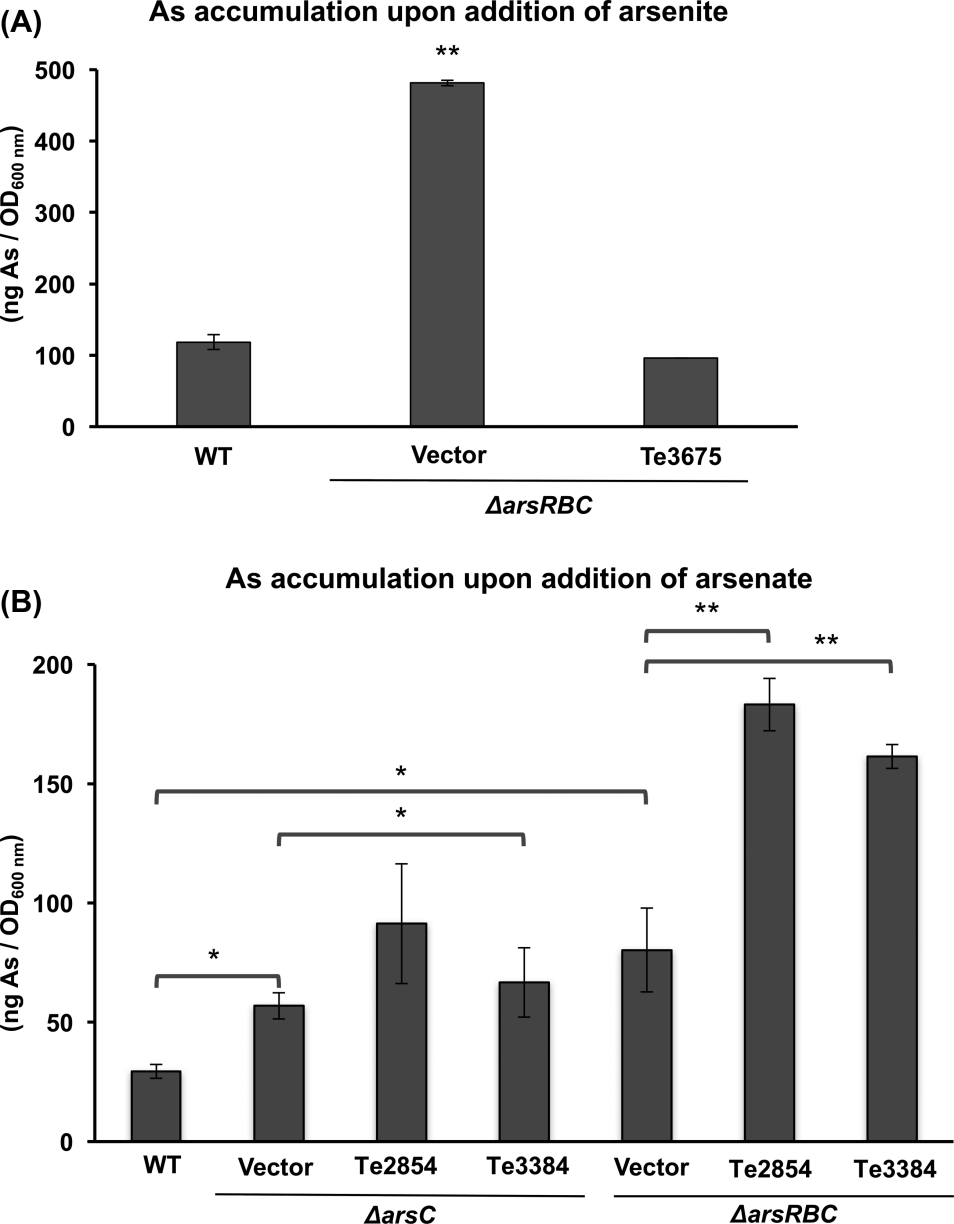
Total arsenic accumulation assays of *E. coli* mutant strains expressing the *ars* homologues. The wild-type strain (WT), and the *∆arsC* and *∆arsRBC* mutant strains with the vector plasmid (Vector) or plasmids expressing the indicated *arsC* (Te2854 and Te3384) or *arsB* (Te3675) homologues were grown in LB overnight, and then 3 mM arsenite **(A)** or 1 mM arsenate **(B)** were added. The strains were further incubated for 5 h. Accumulation of total intracellular arsenic was quantified by ICP-MS/MS. Data shown are the average of three independent experiments ± SEM. Differences were considered to be significant in a Student's t-test: *P* ≤ 0.01 (**), *P* ≤ 0.05 (*).

To fully characterize the *arsB* homologue Te3675, and the members of the ArsC-like family Te3384 and Te2854 of *Terribacillus* sp. AE2B 122, the capacity of different *E. coli* strains to accumulate arsenic was evaluated by exposing them to arsenite (expression of *arsB* homologue) or arsenate (expression of the ArsC-like family members). Expression of the *arsB* homologue Te3675 in the *E. coli ∆arsRBC* mutant reduced the accumulation of arsenic to wild-type levels when cells were grown in the presence of 3 mM arsenite (Fig. 5A). Together with the arsenic resistance assays in *E. coli* and the expression data in *Terribacillus* sp. AE2B 122, these data strongly suggest that Te3675p functions as an arsenite efflux pump.

In order to test the effect of expressing the members of the ArsC-like family of *Terribacillus* sp. AE2B 122 Te2854 and Te3384 on arsenic accumulation in *E. coli*, they were first expressed in the *∆arsC* mutant, with 1 mM arsenate added to the cultures. The *E. coli* strain expressing either Te2854 or Te3384 presented slightly higher levels of arsenic accumulation than *E. coli ∆arsC* harboring only the plasmid vector (Fig. 5B), and all accumulated more arsenic than wild type (*E. coli* W3110). Unexpectedly, when Te2854 and Te3384 were expressed in the *E. coli ∆arsRBC* mutant strain, arsenic accumulation increased 2–2.3-fold compared with levels in the control strain *∆arsRBC* harboring the empty plasmid, and over 3-fold compared with the wild-type strain (Fig. 5B). These experiments, together with the lack of arsenic induction in *Terribacillus*, suggest that neither Te2854 nor Te3384 encode arsenate reductases, but rather that they help minimize the toxic effects of arsenic by other means, one of which could be the chelation of the metalloid (Fig. 5B).

### Arsenate reduction in *E. coli ars* mutants expressing the *Terribacillus* Te3384 and Te2854

**Figure 6.**
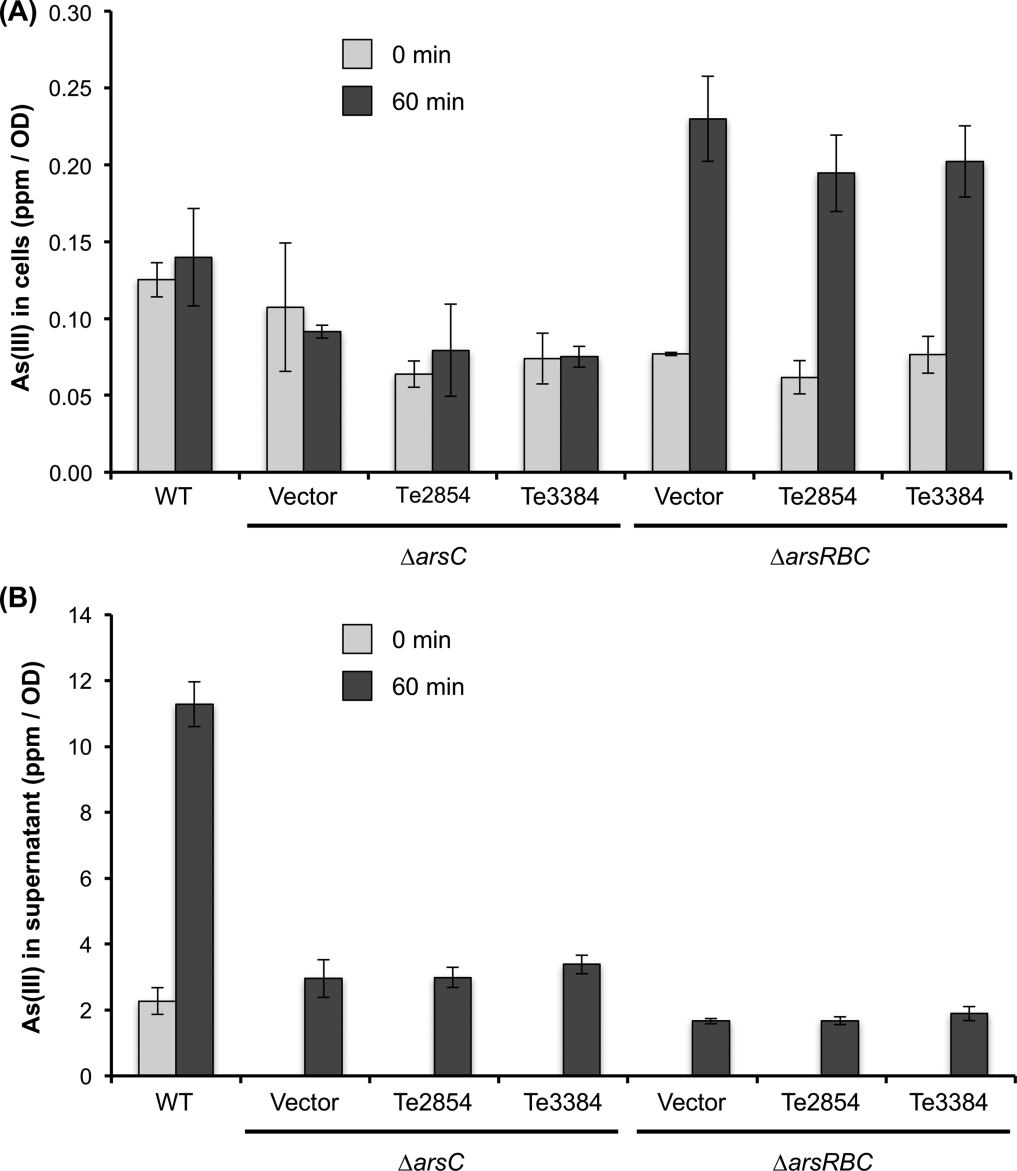
Arsenic speciation in *E. coli* mutant strains expressing Te2854 and Te3384. *Escherichia coli* strains were grown overnight, and then 1 mM IPTG and 1 mM arsenate were added to the cultures. The strains were further incubated for 1 h, and samples were withdrawn. Arsenic speciation was performed from intracellular samples **(A)** and supernatants **(B)** to quantify arsenite. Data shown are the average of three independent experiments ± SEM.

To fully characterize the members of the ArsC-like family Te3384 and Te2854 of *Terribacillus* sp. AE2B 122, the capacity of different *E. coli* strains to reduce arsenate was evaluated by exposing them to arsenate (Fig. 6). After 60 min of incubation of the *E. coli* wild-type strain with 1 mM arsenate, we did not observe changes in the accumulation of arsenite inside the cells. On the other hand, the *∆arsRBC* mutant, which is incapable of pumping arsenite out of the cells, showed an increased accumulation of intracellular arsenite (Fig. 6A). This increased accumulation of arsenite was not observed in the *∆arsC* mutant, which expressed the native arsenite pump, *arsB*. Expression of either Te3384 or Te2854 did not affect the levels of intracellular arsenite in either of the two mutant strains. Furthermore, the amount of arsenite in the supernatant of the cultures after 60 min of incubation with arsenate was 3.3–3.7-fold higher in the *∆arsC* mutant and 5.8–6.5-fold higher in the *∆arsRBC* mutant strains. Expression of the *Terribacillus* Te3384 and Te2854 genes in these mutants did not affect the amount of arsenite in the supernatant of the cultures (Fig. 6B). The most plausible explanation is that the arsenite found inside the cells and in the supernatants originates from ArsC-independent mechanisms, and therefore, taking all these results together, it can be concluded that Te3384 and Te2854 from *Terribacillus* sp. AE2B 122 are not arsenate reductases.

### Role of *Terribacillus* sp. AE2B 122 members of the ArsC-like family in resistance to oxidative stress in *E. coli* strains

Analysis of the domains of Te3384p and Te2854p of *Terribacillus* sp. AE2B 122 predicted that these proteins are members of the TRX-like superfamily (cl00388) and ArsC-like (arsenate reductase) or Spx regulator family (Table S4 and Fig. S1, Supplementary data). The thioredoxin-like superfamily is a large and diverse group of proteins containing a TRX fold that perform different redox reactions. To check whether Te3384 and Te2853 perform any anti-oxidative role under arsenic-derived oxidative stress, we assayed resistance to hydrogen peroxide. Surprisingly, the *∆arsRBC* but not the *∆arsC* mutant of *E. coli* was more sensitive to H_2_O_2_ than the wild-type strain. Interestingly, expression of either Te2854 or Te3384 removed this sensitivity (Fig. S5, Supplementary data).

### Arsenic speciation in *Terribacillus* sp. AE2B 122

**Figure 7.**
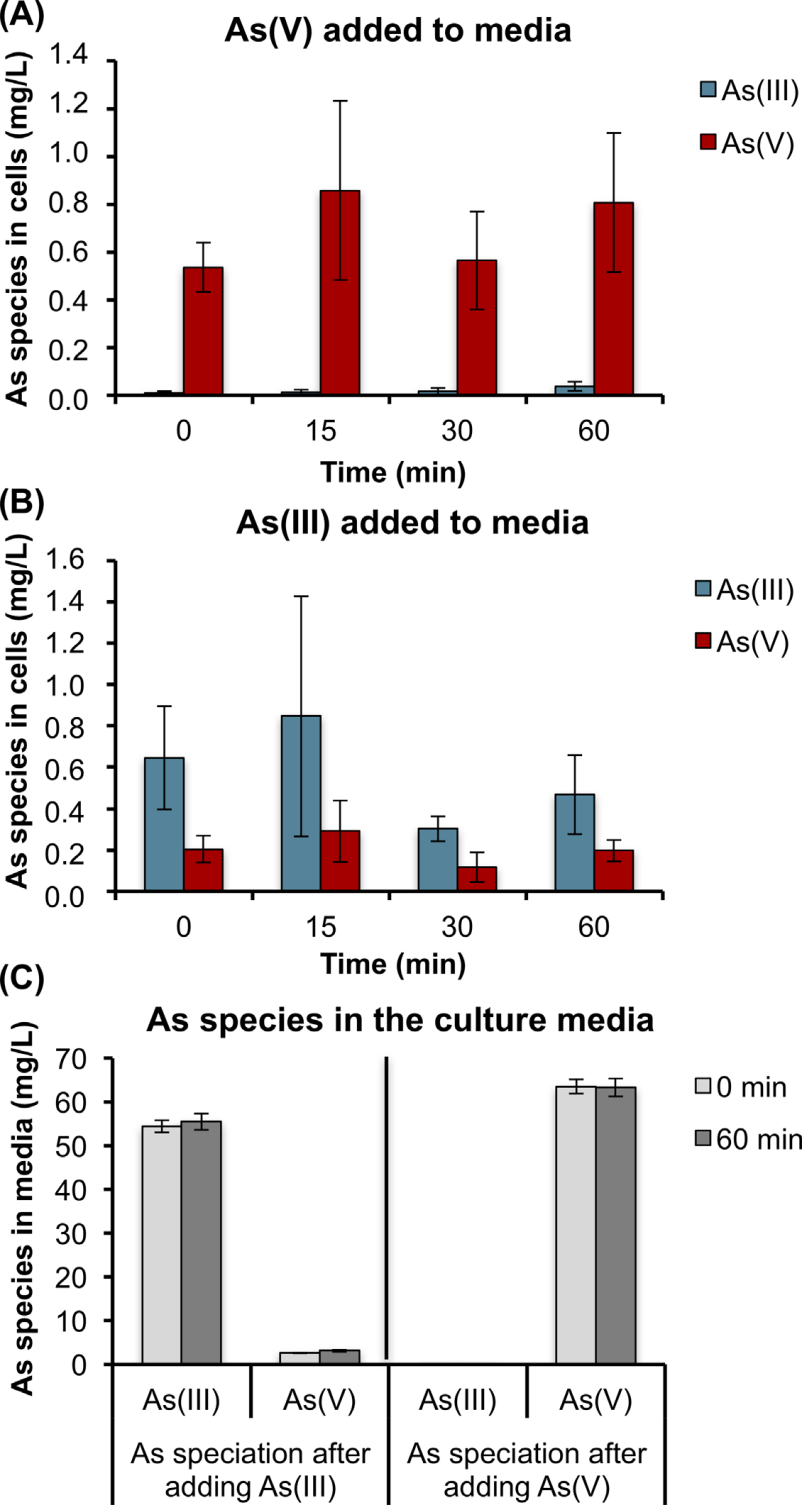
Arsenic speciation in *Terribacillus* sp. AE2B 122. *Terribacillus* was grown overnight, and then 1 mM arsenate **(A, C)** or 1 mM arsenite **(B, C)** were added. The strains were further incubated for up to 1 h, and samples were withdrawn at the indicated times. Arsenic speciation was performed from intracellular samples (A, B) and supernatants (C) to quantify arsenite and arsenate inside the cells (A, B) or pumped out to the culture media (C). Data shown are the average of three independent experiments ± SEM.

The data obtained strongly suggested that neither Te2854 nor Te3384 function as arsenate reductases in *Terribacillus* sp. AE2B 122, and yet this bacterium could tolerate at least 10 mM arsenate. However, since the genome sequence is not complete, we cannot discard the possibility that another member of the ArsC-like family exists in the genome of *Terribacillus*, and that it could play the alleged arsenate reductase role. Therefore, we investigated whether *Terribacillus* sp. AE2B 122 can reduce arsenate by performing arsenic speciation assays. Next, 1 mM arsenite or 1 mM arsenate was added to *Terribacillus* cultures and samples were taken at different time points. Arsenic speciation was performed in the intracellular fractions. When arsenate was added to the media, only arsenate could be detected inside the cells (Fig. 7A). However, when arsenite was added to the media, small amounts of arsenate were also detected inside the cells (Fig. 7B). Since the amount of arsenate did not change over the time course of the experiment and was equivalent to the control sample at time 0, we concluded that the arsenate was probably due to oxidation of arsenite to arsenate in the presence of ambient oxygen, and that it was likely to be present in the commercial powder. Arsenite was not pre-reduced before starting the experiment (Cánovas *et al*. 2003). To rule out the possibility that arsenate is reduced to arsenite, and immediately pumped out of the cell, we also analyzed the arsenite and arsenate content of the supernatant (Fig. 7C). Similar to what we observed inside the cells when arsenite was added to the media, residual amounts of arsenate were found in the supernatant, which remained constant over time. When arsenate was added to the media, only arsenate was detected in the supernatant. Taken together, these results suggest that *Terribacillus* sp. AE2B 122 is incapable of reducing arsenate to arsenite.

## DISCUSSION

*Terribacillus* sp. AE2B 122 is an environmental strain isolated from olive oil agroindustry waste. The resistance assays in liquid medium showed that this microorganism was resistant to up to 2.5 mM arsenite and at least 10 mM arsenate under our assay conditions (Fig. 1). To gain insights into the complex system underpinning the bacterial contribution to the biogeochemical cycling of arsenic, we analyzed the mechanism used by *Terribacillus* sp. AE2B 122 to tolerate this toxic metalloid. The bioinformatics analysis of *Terribacillus* sp. AE2B 122 genome revealed that there are eight ORFs that showed sequence homology to typical arsenic resistance genes: two *arsB-like* genes (Te2733, Te3675); two *arsC*-like genes (Te3384, Te2854); and four *arsR*-like genes (Te2310, Te3140, Te3676, Te3724). Interestingly, a global survey of arsenic resistance genes reported that many microorganisms harbor several copies of *arsB*, and in particular several *Bacillus* strains harbor two *arsB* copies of different clades (Dunivin, Yeh and Shade 2019). Indeed, previous reports showed that *Pseudomonas putida* harbors two *ars* operons, and that both are functional depending on the environmental conditions (Canovas, Cases and de Lorenzo 2003; Paez-Espino, Durante-Rodriguez and de Lorenzo 2015). Our bioinformatics analysis using the RAST server also revealed two genes in the genome of *Terribacillus* that display homology to *arsB*. However, several lines of evidence strongly suggest that only Te3675 is an arsenite efflux pump: (i) it appears in an operon together with a homologue of *arsR* (Te3676); (ii) it is induced in response to arsenate in *Terribacillus* sp. AE2B 122 (Fig. 3); (iii) it promoted arsenite resistance in an *E. coli ∆arsRBC* mutant (Fig. 4); and (iv) it reduced arsenic accumulation in *E. coli ∆arsRBC* grown in the presence of arsenite (Fig. 5). Therefore, this example suggests that functional analyses are essential to determine whether all *arsB* copies of a species are *bona fide* arsenite pumps. It has been suggested that an ancestral *arsRB* operon could have evolved in anaerobic environments during the early stages of the evolution of life, when arsenite was the predominant arsenic oxyanion (Rosen 1999). The existence of this minimal *arsRB* operon was first described in *Ferroplasma acidarmanus*, which presented an additional gene, *arsA*, located separately from the *arsRB* operon (Gihring *et al*. 2003; Baker-Austin *et al*. 2007). However, the functionality of this *arsRB* operon was not demonstrated. In *F. acidarmanus*, homologues of the arsenate reductase *arsC* were not found, indicating that additional, unknown arsenate-resistance genes exist for this organism, as our results indicate could be the case in *Terribacillus* sp. AE2B 122.

Neither Te3384 nor Te2854 were able to fully revert the arsenate sensitivity of a *∆arsC* mutant, they did not reduce arsenate accumulation, and they did not reduce arsenate when they were expressed in *E. coli ∆arsRBC* and *∆arsC* strains. Furthermore, their expression was not induced by arsenic. We cannot rule out the possibility that other genes displaying homology to *arsC* are present in the genome of *Terribacillus* sp. AE2B 122, as the genome sequence is not complete. However, additional arsenic speciation experiments revealed that *Terribacillus* sp. AE2B 122 does not reduce arsenate to arsenite (Fig. 7). Therefore, we can conclude that they are not *bona fide* arsenate reductases. However, both conferred some increased resistance to arsenate in *E. coli Δars* mutants, while accumulation assays showed that *E. coli ΔarsRBC* expressing Te3384 and Te2854 accumulated higher amounts of arsenic (Fig. 5B). Both Te3384p and Te2854p proteins contain predicted domains of the TRX-like superfamily as well as GSH-transferases (GST) and glutaredoxins (GRX). One hypothesis is that both Te3384 and Te2854 mediate formation of GSH-arsenate/arsenite complexes, resulting in increased arsenic accumulation in the cell, like the GRX or the GST reported in *Pteris vittata* (Sundaram *et al*. 2008) or *E. coli*(Chrysostomou*et al*.^2015^). Another hypothesis is that Te3384 and Te2854 contribute to tolerance to oxidative stress (see Fig S5, Supplementary Data). Both proteins contain Spx-related domains, and they appeared closely related to Spx members in the ArsC evolutionary relationship tree. GRX, GST and Spx functions have been associated with protection against oxidative stress (Sharma *et al*. 2004; Zuber 2004; Reder *et al*. 2008; Jozefczak *et al*. 2012; Berndt and Lillig 2017; Sundaram *et al*. 2008). A recent article also reports the role of *arsH* (a NADPH-dependent flavin mononucleotide reductase encoded in the *ars* operons of *P. putida*) in relieving the oxidative stress generated by arsenite and arsenate (Paez-Espino *et al*. 2020).

In conclusion, the environmental strain *Terribacillus* sp. AE2B 122 harbors an unusual and simple *ars* gene cluster. How does arsenic homeostasis work in *Terribacillus* sp. AE2B 122? Arsenite is taken up by the cell and subsequently pumped out of the cell by the ArsB efflux system, resembling what is happening in most bacteria. However, the absence of a *bona fide* arsenate reductase suggests that alternative, yet to be identified mechanisms must operate for resistance to arsenate. Te2854 and especially Te3384 seem to contribute to arsenate resistance, possibly by reducing the oxidative stress generated by arsenic and/or by the formation of arsenic complexes inside the cells.

## Supplementary Material

fiab015_Supplemental_File
