# Supplementary material for: An arsRB resistance operon confers tolerance to arsenite in the environmental isolate Terribacillus sp. AE2B 122
Source: FEMS Microbiol Ecol. 2021 Jan 29;97(3):fiab015. doi: 10.1093/femsec/fiab015 (PMC8755942; doi:10.1093/femsec/fiab015)
Supplement: fiab015_Supplemental_File [file fiab015_supplemental_file.pdf]

SUPPLEMENTARY DATA

An *arsRB* resistance operon confers tolerance to arsenite in the environmental isolate *Terribacillus* sp. AE2B 122

Almudena Escobar-Niño[a,b*], Leyre Sánchez-Barrionuevo[a,b], José Miguel Torres-Torres[a], Rafael Clemente[c], Gabriel Gutiérrez[a], Encarnación Mellado[b] and David Cánovas[a#]

[a]Department of Genetics, Faculty of Biology, University of Seville, Seville, Spain

[b]Department of Microbiology and Parasitology, Faculty of Pharmacy, University of Seville, Seville, Spain

[c] CEBAS-CSIC, Campus Universitario de Espinardo, Murcia, Spain

[*]Pressent address. Andalusian Center for Grape and Grapevine Research (IVAGRO), Microbiology Lab, University of Cadiz, Puerto Real, 11510, Spain

Table S1. ***E. coli*** **strains used in this work**

Bacterial strain	Characteristics	Reference
Escherichia coli **DH5α**	F^-, *lac*ZΔM15, *rec*A1, *hsd*R1, *sup*E44Δ (*lac*ZYA *arg*F) Nal^R	D. Hanahan (1983)_ENREF_17
E. coli **W3110**	K-12 F^- (IN[rrnD-rrnE])	B. J. Bachmann (1987)
E. coli **AW3110**	*E. coli* W3110 (Δ*ars* RBC::*cam* IN[rrnD-rrnE])	A. Carlin et al. (1995)
E. coli **WC3110**	*E. coli* W3110 *ΔarsC* IN[rrnD-rrnE])	Mukhopadhyay et al. (2000)
E. coli **AW3110/pTe2733**	*E. coli ΔarsRBC* with the plasmid pTe2733 expressing a homolog of *arsB* (*Te2733*) from *Terribacillus*	This work
E. coli **AW3110/pTe3675**	*E. coli* AW3110 (Δ*ars*::*cam* IN[rrnD-rrnE] with the plasmid pTe3675 expressing a homolog of *arsB* (Te3675) from *Terribacillus*	This work
E. coli **AW3110/pTe3384**	*E. coli* AW3110 (Δ*ars*::*cam* IN[rrnD-rrnE] with the plasmid pTe3384 expressing a homolog of *arsC* (Te3384) from *Terribacillus*	This work
E. coli **AW3110/pTe2854**	*E. coli* AW3110 (Δ*ars*::*cam* IN[rrnD-rrnE] with the plasmid pTe2854 expressing a homolog of *arsC* (Te2854) from *Terribacillus*	This work
E. coli **AW3110-pTe2733/pTe3384**	*E. coli* AW3110 (Δ*ars*::*cam* IN[rrnD-rrnE] with the plasmids pTe2733 and pTe3384	This work

***E. coli* AW3110-pTe2733/pTe2854**	*E. coli* AW3110 (Δ*ars*::*cam* IN[rrnD-rrnE] with the plasmids pTe2733 and pTe2854	This work
***E. coli* AW3110-pTe3675/pTe3384**	*E. coli* AW3110 (Δ*ars*::*cam* IN[rrnD-rrnE] with the plasmids pTe3675 and pTe3384	This work
***E. coli* AW3110-pTe3675/pTe2854**	*E. coli* AW3110 (Δ*ars*::*cam* IN[rrnD-rrnE] with the plasmids pTe3675 and pTe2854 /	This work
***E. coli* WC3110/pTe3384**	*E. coli* WC3110 (Δ*ars*::*cam* IN[rrnD-rrnE] with the plasmid pTe3384	This work
***E. coli* WC3110/pTe2854**	*E. coli* WC3110 (ΔarsC IN[rrnD-rrnE] with the plasmid pTe2854	This work

Table S2. Primers used to generate the constructs for the heterologous expression of *Terribacillus* sp. AE2B 122 ars homologues in *E. coli*

Primer	5´-Sequence-3´	Amplificated gen of *Terribacillus* sp. AE2B 122
**MAO393**	ACAGAATTCAAAGAGGAGAAATTAAGCATGTTAACAACTGTTC	*arsB*3675
**MAO394**	CCGGGTACCTTAATGGATGATGGATAACCAAATA	
**MAO398**	ACAGAATTCAAAGAGGAGAAATTAAGCATGACATTAGAAATAG	*arsB*2733
**MAO399**	CCGGGTACCCTAACTAAAAACTATTTTGACCCAG	
**MAO160**	TGACAATTAATCATCGGCTCG	pVLT31 and pIZ1016 sequencing
**MAO173**	CGAAGTAATCGCAACATCCGC	pVLT31 sequencing
**MAO403**	ACAGAATTCAAAGAGGAGAAATTAAGCATGTTCCGATTTTATTGG	*arsC*3384
**MAO404**	CGCTCTAGACTAAGCAGTTACAGCCTCGTATGCT	*arsC3384* and pIZ1016 sequencing of ArsC 3384 positive clones
**MAO406**	ACAGAATTCAAAGAGGAGAAATTAAGCATGGTAACACTTTATACC	*arsC*2854
**MAO407**	CGCTCTAGATCAGTTGACCATACGCTGTGCTTCA	ArsC2854 and pIZ1016 sequencing of ArsC 2854 positive clones

Table S3. Primers used for RT-qPCR

Primer	5´-Secuence-3´	*Terribacillus* sp. AE2B 122 gene amplification
**gyrA122F**	AATCGAAAAAGGCGAATGGA	Gyrase (subunit A)
**gyrA122R**	TCCGTTTGGAAATCCCTTGTT	
**rpoB122F**	AGAATATCTTGGAGCCGCATGA	DNA polymerase (subunit β)
**rpoB122R**	TAGTGTACGTTCGCCTTCTGGAT	
**ArsB2733F**	TTGAAGTGGATGGACTATCTCAAAGT	*arsB*2733
**ArsB2733R**	TTTTGACCCAGTAATAGAGTAGGAACAA	
**ArsB3675F**	CCCAAAAAGGTGTCAAAATATCG	*arsB*3675
**ArsB3675R**	GGATAACCAAATATAAAGTCCTGTTAGTGTT	
**ArsC2854F**	GCGTTTGCAGGTAGGCTACAA	*arsC*2854
**ArsC2854R**	CAACTGGAATGTACGCACAGTTC	
**ArsC3384F**	GGAGAAAATCAAAGAGGCAGATG	*ars*C3384
**ArsC3384R**	GACGCTTAATCAGCATACCGTTT	
**ArsR2310F**	ACGTGGAAACAGCTCGAAGAA	*arsR*2310
**ArsR2310R**	TTTCGCTCGCTCCAATCC	
**ArsR3140F**	CCGAGTCATCCTTTGCCAAA	*arsR*3140
**ArsR3140R**	CCAAATGCTCCCAATTGCA	
**ArsR3676F**	GACTTGTGAACAGCACGCTTTG	*arsR*3676
**ArsR3676R**	CCTTACAAGCTGCTCGTCATACTC	
**ArsR3724F**	TCTGGCCATGATGCAACGT	*arsR*3724
**ArsR3724R**	TCTGCGATAATATGTCCATTGGTT	

Table S4. *ars* homologues in the *Terribacillus* sp. AE2B 122 genome.

Genome location (Name)	Product designation	Putative function	Predicted size (aa)	Domain hits	% I with closest relationship[a]
**Scaffold5_3_32527_32132 (Te2854)**	ArsC/Spx	Glutaredoxin-dependent arsenate reductase /transcriptional regulator Spx	131	spxA(PRK01655) ArsC_Spx(cd03032) AsrC(COG1393) ArsC(pfam03960)	131/131(100%) with transcriptional regulator Spx [*Bacillaceae*] (WP_038559379.1)
**Scaffold7_3_9133_9486 (Te3384)**	ArsC/Spx	Glutaredoxin-dependent arsenate reductase /transcriptional regulator Spx	117	ArsC_like(cd03036) ArsC(COG1393) arsC_related(TIGR01617) ArsC(pfam03960)	117/117(100%) with arsenate reductase family protein [*Terribacillus saccharophilus*] (WP_095218350.1)
**Scaffold9_2_5245_3947 (Te3675)**	ArsB	Arsenite efflux pump	432	PRK15445(PRK15445) 2a45(TIGR00935) ArsB(pfam02040) ArsB_permease(cd01118) ArsB(COG1055)	432/432(100%) with arsenic transporter [*Terribacillus saccharophilus*] (WP_095220793.1)
**Scaffold4_6_24396_23038 (Te2733)**	ArsB	Arsenite efflux pump	452	ArsB_permease(cd01118) ArsB(COG1055) PRK15445(PRK15445) 2a45(TIGR00935) ArsB(pfam02040)	449/452(99%) with arsenic transporter [*Terribacillus saccharophilus*] (WP_095218693.1)
**Scaffold3_3_113452_114177**	ArsR	DNA-binding	241	HTH_ARSR(cd00090)	237/241(98%) with ArsR

**(Te2310)**		transcriptional regulator, ArsR family		HTH_ARSR(smart00418) ArsR(COG0640) HTH_20(pfam12840)	family transcriptional regulator [*Terribacillus saccharophilus*] (WP_095271968.1)
**Scaffold6_4_20014_20634 (Te3140)**	ArsR	DNA-binding transcriptional regulator, ArsR family	206	HTH_ARSR(cd00090) HTH_20(pfam12840) ArsR(COG0640) HTH_ARSR(smart00418)	206/206(100%) with ArsR family transcriptional regulator [*Terribacillus saccharophilus*] (WP_095269594.1)
**Scaffold9_2_5609_5265 (Te3676)**	ArsR	DNA-binding transcriptional regulator, ArsR family	114	HTH_ARSR(cd00090) HTH_5(pfam01022) HTH_ARSR(smart00418) ArsR(COG0640) PRK10141(PRK10141)	114/114(100%) with winged helix-turn-helix transcriptional regulator [*Terribacillus saccharophilus*] (WP_095220794.1)
**Scaffold9_5_34304_33990 (Te3724)**	ArsR	DNA-binding transcriptional regulator, ArsR family	104	ArsR(COG0640) HTH_ARSR(cd00090) HTH_ARSR(smart00418) PRK10141(PRK10141) HTH_20(pfam12840)	104/104(100%) with MULTISPECIES: helix-turn-helix transcriptional regulator [*Terribacillus*] (WP_038565330.1)

[a] I, identity, with sequences from closest proteins in Blastp results. Access numbers are shown in parentheses.

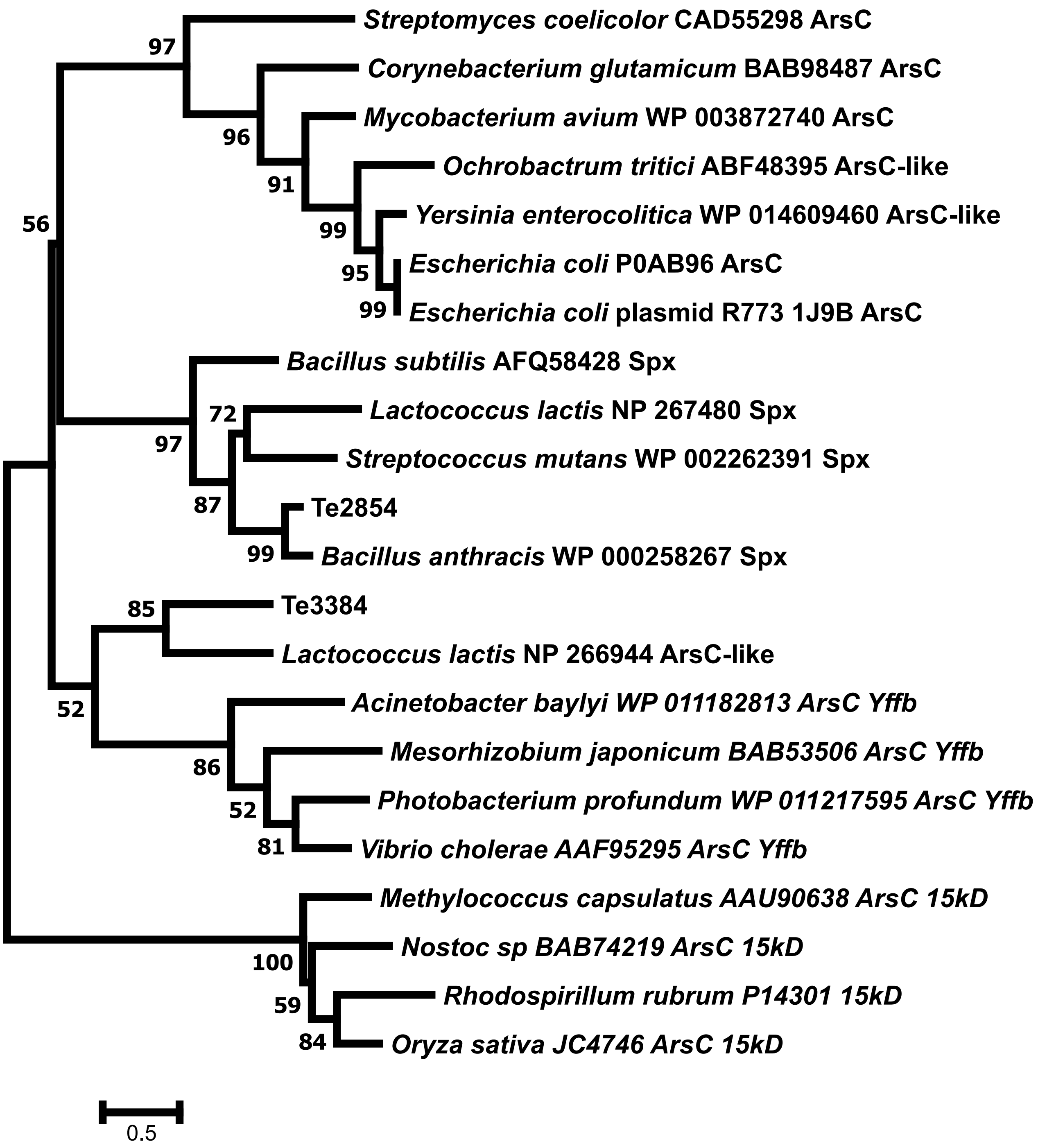

Figure S1. **Phylogenetic reconstruction of arsenate reductases homologues**. The tree shows the phylogenetic relationships of 20 representative proteins of the five subfamilies included in the cd02977 ArsC_family and the ArsC homologues (Te3384p and Te2854p) of *Terribacillus* sp. AE2B. Multiple alignments were made with Clustal Omega. Maximum-likelihood tree was generated using the IQ-Tree server. Branch support analysis was evaluated by 1 000 ultrafast bootstrap replicates. ModelFinder was used to find the free rate heterogeneity substitution model that best fit the alignment and on line TOl was used for tree editing.

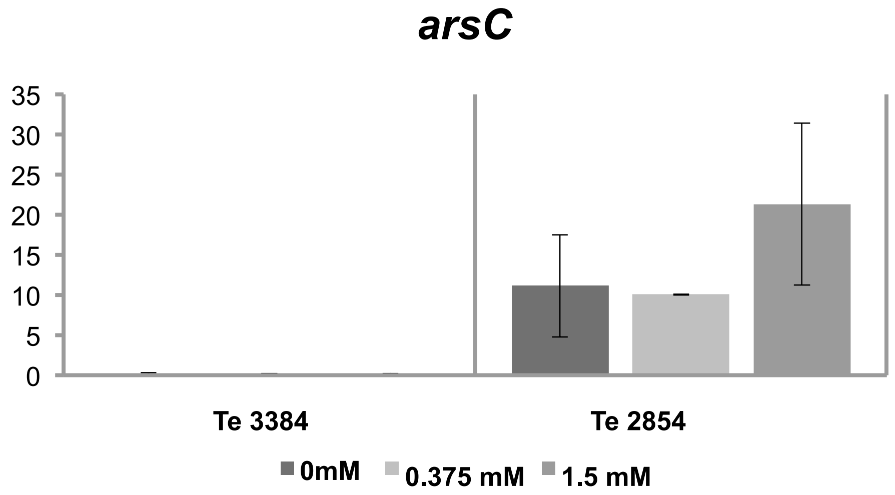

Figure S2. Gene expression of *Terribacillus* sp. AE2B 122 *arsC* homologues in response to arsenate induction. *Terribacillus* was grown in PYA and induced for 6 hours with 0 mM, 0.375 mM or 1.5 mM arsenate. The expression of the *arsC* homologues (Te3384 and Te2854) was quantified by RT-qPCR and normalized to *gyrA* expression. Gen expression without relativization is represented.

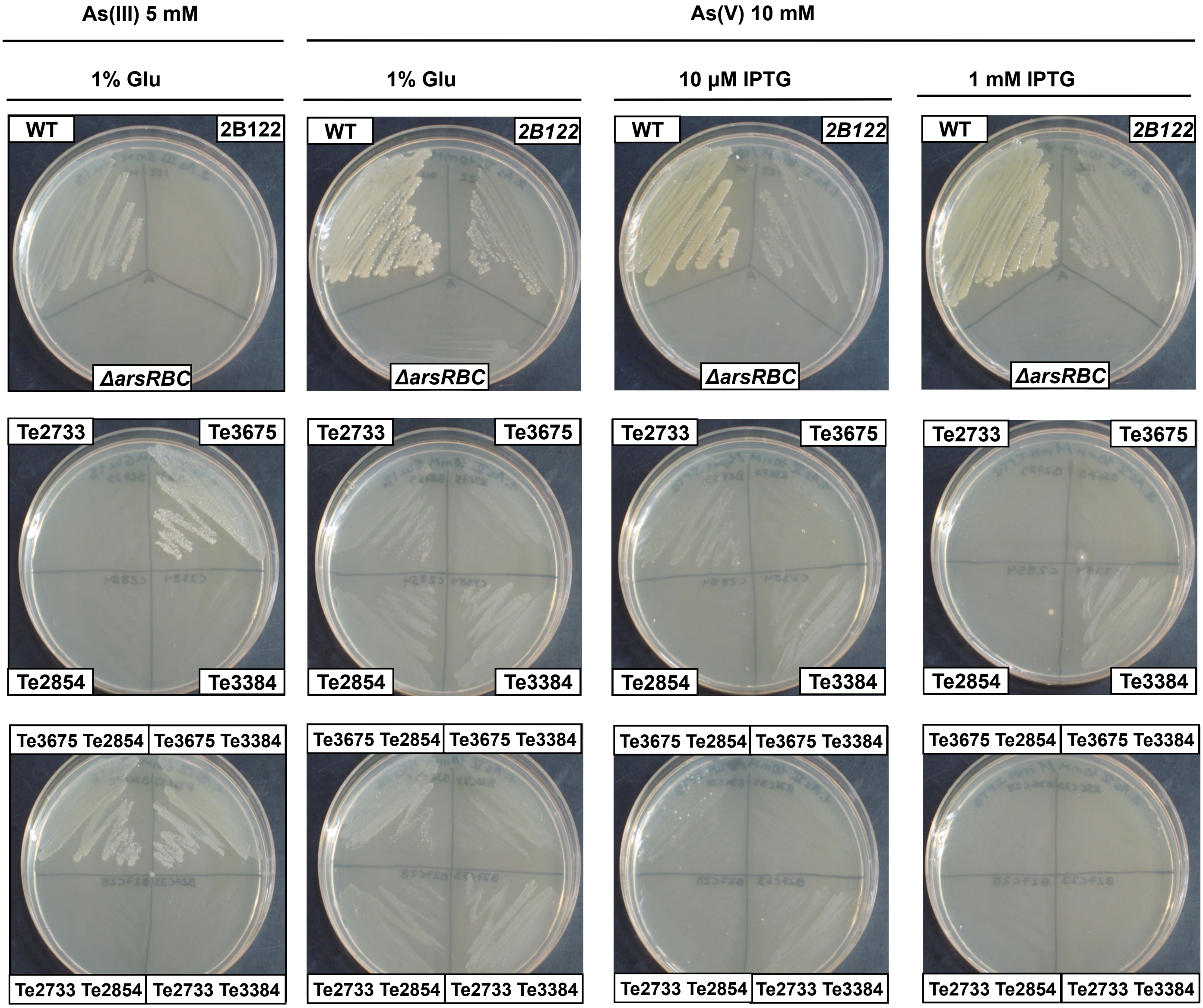

Figure S3. Arsenic resistance assays of *E. coli* Δ*arsRBC* strains expressing *Terribacillus* sp. AE2B 122 *ars* homologues. *Terribacillus* sp. AE2B 122 (2B122), *E. coli* W3110 (wild type; WT) and *E. coli* AW3110 (*ΔarsRBC*) expressing the *arsB* homologues (Te2733 and Te3675) and/or the *arsC* homologues (Te3384 and Te2854*)* in different combinations were inoculated on PYA+1.5% NaCl solid medium with 5 mM arsenite, As(III), or 10 mM arsenate, As(V), and 1% glucose (Glu), 10 µM IPTG or 1mM IPTG. Plates were photographed after 3 days of growth at 30ºC.

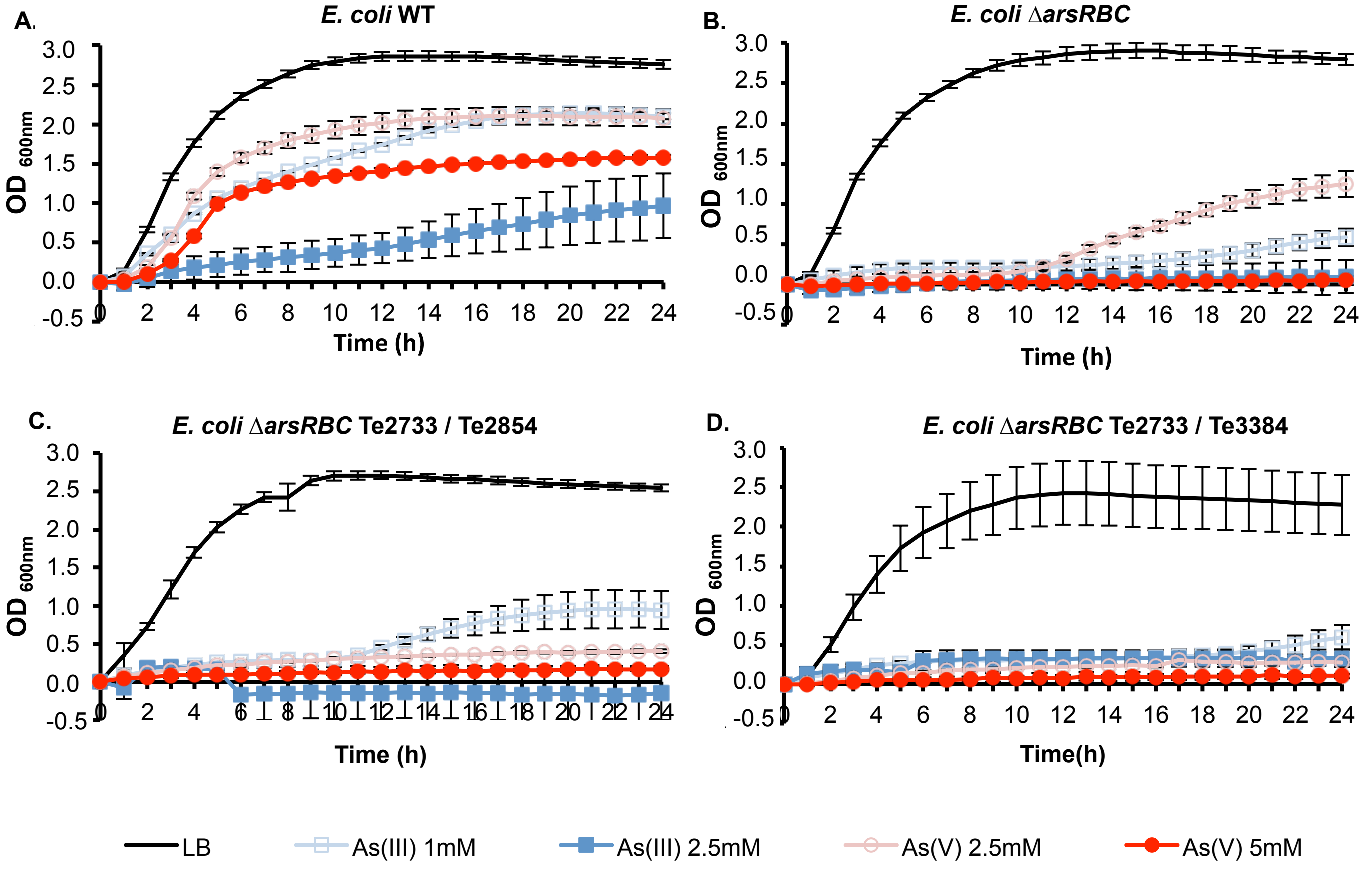

Figure S4. Arsenic resistance assays of *E. coli* W3110 and *E. coli ΔarsRBC* strains expressing *Terribacillus* sp. AE2B 122 *arsB/arsC* homologues. *E. coli* W3110 (wild type; *E. coli* WT) and *E. coli* AW3110 (*E. coli ΔarsRBC*) expressing the *arsB* homologue Te2733 in combination with *arsC* homologues (Te3384 or Te2854*)*. The strains were inoculated in LB medium with 1 mM arsenite, As(III); 2.5 mM arsenite, As(III); 2.5 mM arsenate, As(V); or 5 mM arsenate, As(V) at 37ºC. Data are the mean of at least three independent experiments performed in duplicates. SEM bars are not depicted for clarity.

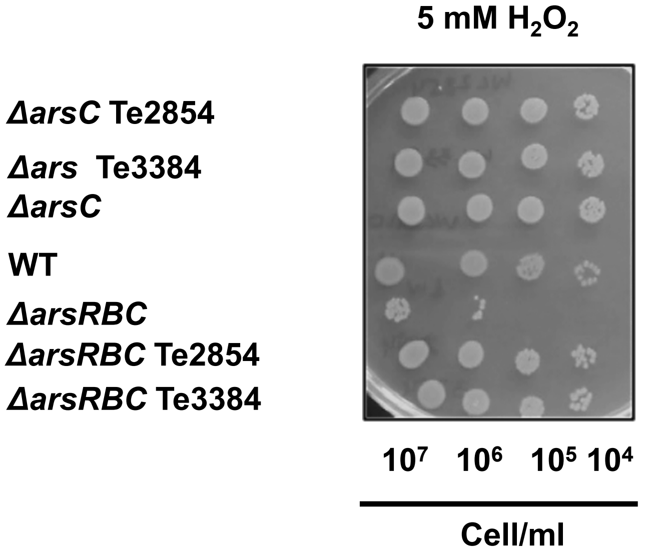

Figure S5. **Oxidative stress resistance assay using LB solid medium with H_2O_2**. Serial dilutions of the different *E. coli* strains were spotted on LB medium containing 5 mM H_2O_2 and 1 mM IPTG. *E. coli* W3110 (WT), *E. coli* AW3110 (*ΔarsRBC*), *E. coli* WC3110 (*ΔarsC*) expressing one of the *arsC* homologues (Te3384 and Te2854). Plates were photographed after 2 days of growth at 37ºC.
